# Altered phosphate metabolism in HIV-1-infected patients: another feature of metabolic syndrome?

**DOI:** 10.1186/1758-2652-13-S4-P78

**Published:** 2010-11-08

**Authors:** P Grima, A Zizza, M Guido, P Calabrese, R Chiavaroli, MR Sodo, M Tana, P Tundo

**Affiliations:** 1 S.Caterina Novella, Infectious Diseases, Galatina, Italy; 2 National Research Centre, Clinical Physiology Institute, Lecce, Italy; 3 Faculty of Sciences, University of Salento, Biological and Environmental Sciences and Technolo, Lecce, Italy

## Purpose of the study

Metabolic syndrome represent a cluster of cardiovascular risk factors that has become a serious problem for HIV-1-infected patients. It was proposed that disturbances in phosphate metabolism may represent a key feature of metabolic syndrome. Because phosphate is involved directly in carbohydrate metabolism, hypophosphatemia can results in impaired utilization of glucose, insulin resistance and hyperinsulinemia. Thus, we undertook the present study to investigate the relationship between phosphate levels and the presence of the characteristics of metabolic syndrome, as well as the mechanism that may be responsible for reduced phosphate levels in patients with this syndrome.

## Methods

130 HIV-1-infected patients were consecutively enrolled in a prospective, cross-sectional, single centre study. All patients were receiving HAART for more than six months. We selected two groups: HIV+ patients with metabolic syndrome (group A, n=86) and HIV+ patients without metabolic syndrome (group B, n=44). The diagnosis of metabolic syndrome was based on Adult Treatment Panel III guidelines. Demographic characteristics, metabolic variables, duration of Tenofovir therapy, duration of HAART, CD4 and viral load were collected. Kidney tubular function was examined using tubular resorption of phosphate and normalized renal threshold phosphate concentration.

## Summary of results

**Figure 1:**
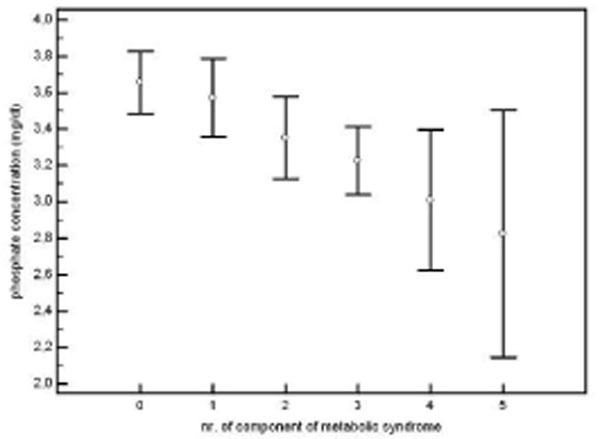


Patients with metabolic syndrome showed significantly lower phosphate (3.13 mg/dl vs 3.55 mg/dl, p<0.01) and higher insulin (13.2 mg/dl vs 6.9 mg/dl, p<0.01) levels compared with controls. There was a linear significant decrease in phosphate values as the number of components of metabolic syndrome increased (p<0.001). Multiple regression analysis including all 5 components of metabolic syndrome and months of TDF treatment showed that insulin level was the most discriminant of serum phosphate (r= -0.22, p<0.01). Figure 1

## Conclusions

Our preliminary data demonstrated that HIV-1-infected patients with metabolic syndrome showed significantly lower phosphate levels compared with HIV-1-infected patients without metabolic syndrome regardless of tenofovir based therapy. The clinical significance of these disturbances, as well as their importance as target for preventive or therapeutic interventions, remains to be established.

